# Development of a Calcium Phosphate Nanocomposite for Fast Fluorogenic Detection of Bacteria

**DOI:** 10.3390/molecules190913948

**Published:** 2014-09-05

**Authors:** Claudio R. Martínez, Tamara L. Rodríguez, Raisa Zhurbenko, Ivonne A. Valdés, Sávio M. L. Gontijo, Alinne D. M. Gomes, Diego F. Suarez, Rubén D. Sinisterra, Maria E. Cortés

**Affiliations:** 1 Centro Nacional de Biopreparados, Carretera a Beltrán Km 1 1/2, Bejucal, Mayabeque, Apartado 6048, Cuba; E-Mails: claudio@biocen.cu (C.R.M.); lobaina@biocen.cu (T.L.R.); raisa@biocen.cu (R.Z.); ivonne.alfonso@biocen.cu (I.A.V.); 2 Restorative Dentistry Department, Faculty of Dentistry, Universidade Federal de Minas Gerais, Av. Antônio Carlos 6627, CEP 31270-901 Belo Horizonte, MG, Brazil; E-Mail: savio.morato@yahoo.com.br; 3 Chemistry Department, ICEx, Universidade Federal de Minas Gerais, Av. Antônio Carlos 6627, CEP 31270-901 Belo Horizonte, MG, Brazil; E-Mails: alinnedamasia@gmail.com (A.D.M.G.); diego.fersuar@gmail.com (D.F.S.); sinisterra@ufmg.br (R.D.S.)

**Keywords:** hydroxyapatite, bacterial adhesion, nanoparticle, nanocomposite, fluorescence

## Abstract

Current procedures for the detection and identification of bacterial infections are laborious, time-consuming, and require a high workload and well-equipped laboratories. Therefore the work presented herein developed a simple, fast, and low cost method for bacterial detection based on hydroxyapatite nanoparticles with a nutritive mixture and the fluorogenic substrate. Calcium phosphate ceramic nanoparticles were characterized and integrated with a nutritive mixture for the early detection of bacteria by visual as well as fluorescence spectroscopy techniques. The composite was obtained by combining calcium phosphate nanoparticles (Ca:P ratio, 1.33:1) with a nutritive mixture of protein hydrolysates and carbon sources, which promote fast bacterial multiplication, and the fluorogenic substrate 4-methylumbellipheryl-β-d-glucuronide (MUG). The composite had an average particle size of 173.2 nm and did not show antibacterial activity against Gram-negative or Gram-positive bacteria. After an *Escherichia coli* suspension was in contact with the composite for 60–90 min, fluorescence detected under UV light or by fluorescence spectrophotometer indicated the presence of bacteria. Intense fluorescence was observed after incubation for a maximum of 90 min. Thus, this calcium phosphate nanocomposite system may be useful as a model for the development of other nanoparticle composites for detection of early bacterial adhesion.

## 1. Introduction

Current culture based procedures for the detection and identification of bacterial infections are laborious, time-consuming, and require a high workload and well-equipped laboratories [1]. Recently, a polymerase chain reaction method was developed which reduced the limit of detection of different microorganisms in foods and increased the speed of their identification [2]. This technique is also being widely applied in clinical laboratories for bacterial detection in human samples due to the reduced costs of reagents and equipment [3]. In addition, chromogenic and fluorogenic methods have been developed for faster and more accurate detection and identification of bacteria and yeast; these methods can provide data in a shorter time with higher accuracy (95%–100%) than those based on current methods [4,5].

Nanomaterials are gaining in relevance in the field of microbial diagnostics. They have the ability to absorb or bind a wide variety of chemically defined compounds or biological molecules. Nanospheres, nanotubes, nanofibers, nanoshells, and quantum dots, among others, have helped to reduce the detection time, decrease the detection limit, and increase the diagnostic accuracy [6,7]. These nanomaterials and nanocomposites are mostly based on gold, other metal nanoparticles, ceramics, polymers, monoclonal antibodies or RNA and DNA fragments coupled with immunofluorescent or immunobioluminiscent dyes [6,8,9]. However, some of new diagnostic procedures involve complex methods for manufacturing the nanomaterials, employ highly specific reagents (e.g., monoclonal antibodies, DNA fragments), and require sophisticated equipment for detecting the signals [10]. A new method based on a two-photon luminescence system with gold nanoparticles conjugated with oligopeptides was able to detect a bacterial concentration as low as 10^6^ spores/mL [9].

Methods based on the combination of nanoparticles with fluorescent, chromogenic, or bioluminescent dyes have emerged as cost-effective, accurate, easy to use, and fast alternatives for bacterial detection, enumeration, and identification, particularly in samples with a high or medium bacterial load.

Currently, special attention has been given to the development of ceramic nanocomposites for microbiological applications, mainly as antimicrobials [11,12]. For example, nanohydroxyapatite has been tested as an antimicrobial drug carrier in several structures, alone [13], or as a nanocomposite with polymers, and as a carrier of nanosized metal particles [14].

Hydroxyapatite (HA) and other calcium phosphate ceramics have been widely used as biocompatible ceramic nanomaterials owing to their extremely high surface area, adaptable topography, high bioactivity, high catalytic activity, and elevated absorption and adsorption capacity [15]. The favorable cell adhesion properties of HA are attributed to its adhesion abilities to cell proteins and to its high absorption capacity of different molecules due to its significant surface activity and superficial area to volume ratio [16,17].

In the present work, calcium phosphate ceramic nanoparticles were characterized and proof-of-concept experiments were carried out in order to develop a simple, fast, easy to perform, and low cost method for bacterial detection by combining HA nanoparticles with a nutritive mixture and the fluorogenic substrate 4-methylumbellipheryl-β-d-glucuronide (MUG) specific for *Escherichia coli*. For producing at laboratory scale the composite, a nutritive mixture and the MUG substrate was absorbed onto the HA ceramic particle agglomerates and then dehydrated. The composite was also physicochemically characterized, and its functionality was tested.

## 2. Results and Discussion 

### 2.1. Loss on Drying and Loading Capacity of Hydroxyapatite (HAP-S)

The results of the average loss on drying determination of the HAP-S nanoparticles before submersion in deionized water, as well as the loading capacity (LC) values at 1, 2, and 3 h are shown in Table 1. HAP-S was able to absorb approximately 29% of its weight in water within 1–3 h. Analysis of variance (*n* = 3, *p* < 0.05) showed significant differences between values at different times* versus* the original loss on drying value. A Tukey *post-hoc* test revealed no significant differences at *p* < 0.05 in the LC between 1 and 3 h.

**Table 1:** Loss on drying of the HAP-S and its loading capacity when imbibing from 1 to 3 h in water at 25 °C.

Parameter	Time (h)	Mean and Std. Dev.
Loss on drying (%)	0	1.43 ± 0.60
Loading capacity (%)	1	27.94 ± 1.84
	2	30.21 ± 4.41
	3	29.51 ± 1.40

The average loss on drying/humidity determination results of the HAP-S nanoparticles is coincident with those values reported by other authors for hydroxyapatite structures [18]. The water LC (~29%) presumes an adequate absorption capacity for the nutritive and fluorogenic substrate mixture (chromogenic-fluorogenic liquid- CCL), indispensable for providing the necessary amount of substrate able to be detected by *E. coli* glucuronidase.

### 2.2. pH Determination

The pH value of the HAP-S nanoparticles was close to neutrality (7.2), while a pH value of 6.8 was found for CCL, as expected, which corresponds to that of CromoCen CCL, a chromogenic-fluorogenic liquid medium used for the detection of *E. coli* and coliform bacteria in water samples [19].

The pH (7.2) of the HAP-S nanoparticles can be considered as appropriate for formulating the HAP-S/CCL composite; this pH value is close to that reported by Saleeb and Debruyn [20] for the point of zero surface charge. Moreover, as previously reported, the speed and intensity of fluorogenic substrate (MUG) cleavage were pH dependent; at values less than 5, the substrate cleavage was delayed and the fluorescence intensity decreased faster than at higher pH values [4].Thus, this composite should not negatively influence the bacterial growth or the activation of *E. coli* enzymatic activity.

### 2.3. Characterization of HAP-S and HAP-S/CCL

**Figure 1:**
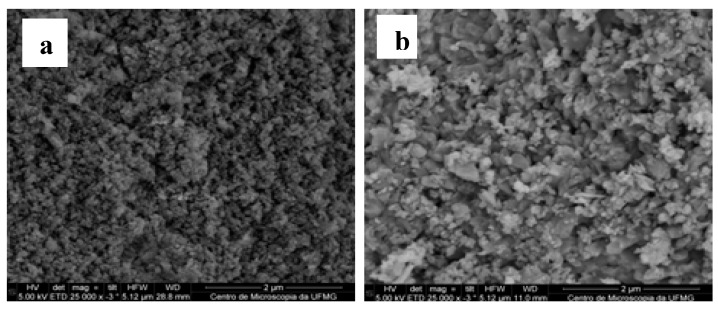
SEM images (**a**) Micrograph of an aggregate of HAP-S nanoparticles confirming that the material is porous. (**b**) HAP-S/CCL nanocomposite containing the nutritive mixture and the fluorogenic substrate showing the particles aggregated. CCL nanoparticles are well integrated in the HAP-S structure and some nanoparticles of this nutritive composite (more light particles) are attached and distributed on the surface of the calcium phosphate matrix.

The HAP-S structure, size, surface, and particle shape analyzed by SEM (Figure 1a) were similar to those of artificially fabricated HA nanoparticles [21] as well as a natural and synthetic HA [22,23]. The aggregates have a significant rough surface topography, enabling the exposure of a high surface contact area. Figure 1a illustrates the SEM images of the HAP-S nanocrystal aggregates, showing a rod shaped form with irregular borders. The images show the nanometric size of individual particles with a highly rough surface.

HA is known as an effective support for proteins, peptides, and bacterial adhesion [24]. Although the mechanism of bacterial adhesion on nanomaterial surfaces is not fully established, studies have demonstrated that major factors are the surface electrical charge, surface topography, and chemical composition [25]. The lack of knowledge on such factors limits the development of more efficient diagnostic systems based on bacteria-nanostructure interactions [26]. In this study, micrographs also showed that the HAP-S nanoparticles (Figure 1a) possess large amounts of nano- and mesosized pores with different diameters, enabling absorption of a significant volume of the CCL solution and consequently supplying the necessary amount of nutrients and fluorogenic substrate to the cells. These properties were confirmed by images of the HAP-S/CCL composites (Figure 1b) and should guarantee a significant contact surface area for bacterial cell attachment. The CCL particles are mostly integrated into the HAP-S support material, fully covering its structure and increasing the particle size without affecting the final roughness of the surface. Some CCL aggregates with a higher mesometric size were exposed on the surface of the composite.

The particle size distributions and the ζ-potentials of the nanosized elemental particles of CCL, HAP-S, and the HAP-S/CCL composite are illustrated in Table 2. As table shows, the nutritive and fluorogenic mixture (CCL) has a bimodal distribution of particle sizes with a minor peak at 25.6 nm (12.7%) and a major peak at 113.2 nm (87.3%). HAP-S is characterized by a definite particle size with a tight monomodal distribution peak with a mean value of 209.0 nm and a negative ζ-potential value of −20.18 ± 0.54 mV. The HAP-S/CCL composite fabricated from the two preceding components showed a broad monomodal particle size distribution with a mean value of 173.2 nm; in addition, its ζ-potential (−20.66 ± 0.54 mV) did not vary significantly when compared with that of the original HAP-S nanoparticles.

**Table 2:** Mean particle size, mean particle size percentage distribution and ζ-potential of tested materials.

Sample	Peak 1		Peak 2		ζ Potential (mV)
	Mean Particle Size (nm)	%	Mean Particle Size (nm)	%	
CCL	113.2	87.3	25.6	12.7	87.3
HAP-S	209.0	100.0	-	-	−20.20
HAP-S/CCL	173.2	100.2	-	-	−20.20

It is now known that the basic chemical composition of HA and calcium phosphate nanoparticles contains some water entrapped in the structure and often includes ions such as Na^+^, K^+^, Mg^2+^ (for Ca^2+^), CO_3_^2−^ (for PO_4_^3−^ and for OH^−^), F^−^, and Cl^−^ (for OH^−^) [27].

It is also known that Ca^2+^, PO_4_^3−^, and OH^−^ ions are the ones that contributes more to the surface charge characteristics (*i.e.*, ζ-potential) of HA; and its value may experience variations when solutions containing anions or cations are added to HA particles [20,28]. The ζ-potential of the HAP-S nanoparticles was negative (Table 2); in addition, most bacteria show overall negative charge in their membrane surfaces. On the other hand, for HAP-S, a negative mobility is observed in the pH range from 5 to 8 [22]. The resulting composite (HAP-S/CCL) showed a ζ-potential value close to that of the HAP-S nanoparticles (Table 2; ζ-potential value = −20.20). *E. coli*, being a Gram-negative bacterium, shows negative charge on its membrane surface, thus any growth promoted by the HAP-S/CCL composite would be based mainly on the chemical surface composition, topography roughness [25], availability of nutrients, and induction of enzymatic activity by CCL components. If any cell-surface interaction occurs, it also may be explained by the production of certain extracellular polymeric substances and other outer membrane proteins in *E. coli* pili [26].

The surface area of particles available for bacteria to react depends on the mean size and the size range of particles [29]. The particle size of the nutritive composite (CCL) used in these experiments (Table 2) to promote bacterial multiplication and to obtain the HAP-S/CCL composite was not homogenous due to the different characteristics and sizes of the components. During medium fabrication at an industrial scale, components such as salts are ground and sieved to a certain particle size (e.g., 0.125 mesh sieves), but other constituents such as protein hydrolysates, fluorogenic substrates, and carbohydrates are not ground in order to avoid denaturation. These components show different solubilities at 37 °C and may not be completely solubilized, but rather well suspended. On the other hand, the average size of the elemental (not aggregated) HAP-S particles (209.0 nm) was twice that of the CCL particles; however, they had a more homogeneous distribution. Both components of the HAP-S/CCL composite (CCL and HAP-S) were integrated in such a form that the average particle dimensions did not increase in size (173.2 nm).

Different hypotheses were formulated to explain this finding: first, almost all of the nutritive composites and culture media formulated with peptones and protein hydrolysates have a high hygroscopicity and solubility; second, the different salts and other chemical substances interact with phosphate or calcium ions of the HAP-S, thus helping to disaggregate the HAP-S particles.

### 2.4. Antibacterial Activity of HAP-S

**Figure 2:**
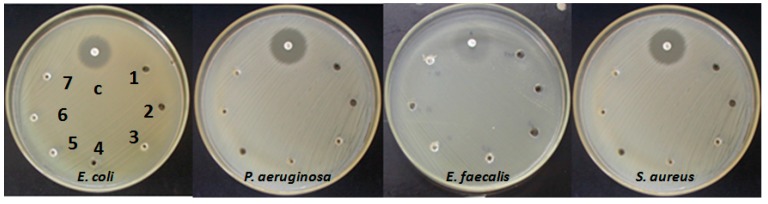
Antimicrobial effect of nanosized particles (HAP-S) at different concentrations (clockwise): 0.05 (1); 0.5 (2); 1 (3); 5 (4); 10 (5); 15 (6) and 20 (7) % w/v against: *E. coli* (ATCC 25922), *P. aeruginosa* (ATCC 27853), *E. faecalis* (ATCC 29212) and *S. aureus* (ATCC 25923) on Mueller- Hinton Agar. Upper center spots (c) show inhibition halos against gentamicin, amikacin, sulfamethoxazole-trimethoprim and chloramphenicol respectively, as positive controls.

The antimicrobial effect of nanosized particles (HAP-S) at different concentrations against *E. coli*,* P. aeruginosa*,* E. faecalis*, and *S. aureus* is illustrated in Figure 2. The antibacterial test results (Figure 2) confirmed the absence of *E. coli*, * P. aeruginosa*,* E. faecalis*, and *S. aureus* inhibition in Mueller-Hinton Agar (MHA) when different amounts of HAP-S nanoparticles were added. The spots numbered from 1 to 7 correspond to 0.1 mL of HAP-S/water suspensions at different concentrations (0.05%, 0.5%, 1%, 5%, 10%, 15%, and 20% w/v, respectively). No inhibition was detected after incubation at 35 ± 2 °C for 24 h, however the inhibition halos of control antibiotics showed their characteristic diameters: 26.5 mm for gentamicin* vs.* *E. coli* and for sulfamethoxazole-trimethoprim* vs.* *E. faecalis*, 24 mm for amikacin* vs.* *P. aeruginosa*, and 28 mm for chloramphenicol* vs.* *S. aureus.*

In some cases, nanoparticles can show antimicrobial activity due to different mechanisms. For example, the smaller particle sizes can break through the cell membrane, causing severe structural damage in the cytoplasm and finally killing the bacterial cell. According to Karlsson* et al.* [30], it cannot be generalized that nanoparticles *per se* are always more toxic than micrometer-sized particles with the same chemical composition. Highly reactive species in the nanoparticle surface may damage peptides, proteins, DNA, and membrane components by oxidation. Other mechanisms of damage have been discussed. For example, high surface to volume ratios and chemical and physical properties can interrupt energy transduction and inhibit enzymatic activity and DNA synthesis [30,31]. None of these inhibition mechanisms seem to be present in current experiments demonstrating the absence of antibacterial activity by HAP-S nanoparticles.

### 2.5. Nutritive Mixture and Bacterial Fluorescence Detection

In a first series of experiments testing different ceramics and calcium phosphate composites it was demonstrated that HAPS-S/CCL shortened the fluorescence detection period for *E. coli*, inoculated at high concentrations of cell suspensions, from 5 h to 2.30 h when comparing it with the exposure of the *E. coli* suspension in CCL alone without the HAP-S [32]. The selection of HAP-S to conform the HAPS-S/CCL nanocomposite was based on the hypothesis that these calcium phosphate aggregates provide a high surface of contact for the enzymatic MUG splitting reaction by bacterial glucuronidase. On the other hand phospahates present in calcium phospahates ceramics are common components of chromogenic and fluorogenic media for growing bacteria [4,5], being essential elements of their enzymatic activity. As previously discussed in Section 2.3, the tested nanocomposite also can guarantee a high CCL volume load providing essential nutrients and markers for *E. coli* and a rough surface for cell attachment, concentrating bacterial cells around the particles and allowing a faster detection of fluorescence reaction.

We evaluated the role of the inclusion of a highly nutritive composite (CCL) as a means to promote fast growth of bacteria and to guarantee the detection of bacteria by fluorescence, at high cell concentrations, without dramatically affecting other material characteristics such as roughness and porosity. The modification of nanoparticles and composites is a common procedure for increasing the detection signal and fastening the detection reaction [9].

**Figure 3:**
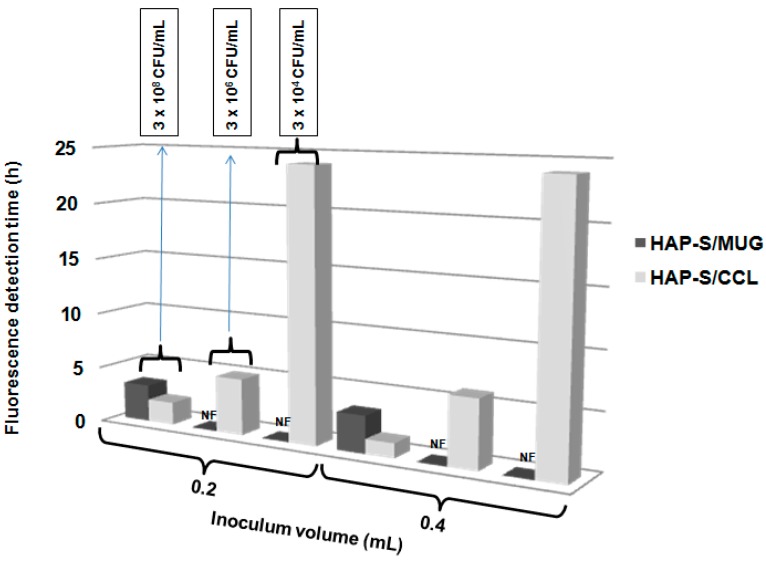
Influence of different composites (HAP-S/CCL and HAP-S/MUG) and inoculum volume (0.2 and 0.4 mL) on fluorescence detection time. NF: no fluorescence was observed after 24 h incubation.

The role of the nutritive mixture on the visual detection time of fluorescence for different *E. coli* concentrations is well illustrated in Figure 3. The presence of the nutritive mixture in the composite (HAP-S/CCL) reduced the fluorescence detection time from 3.5 h (detected with HAP-S/MUG) to 1.5 h, when 0.4 mL of the *E. coli* suspension (3 × 10^8^ CFU/mL) was added. Furthermore, when the inoculum volume was decreased to 0.2 mL at the same concentration, the detection time decreased from 3.5 to 2 h, with an intense fluorescence. For the rest of the tested inoculum concentrations, the HAP-S/MUG composite did not allow visual fluorescence detection even after incubation for 24 h. At a concentration of 3 × 10^6^ CFU/mL, the HAP-S/CCL allowed intense fluorescence to be visible under UV light after incubation for 5–6 h (with 0.2- and 0.4-mL inoculum volumes, respectively). Intense fluorescence was observed even after a lower concentration of *E. coli* was inoculated and incubated for 24 h.

Recently, CdSe/ZnS/SiO_2_ composite nanoparticles were tested as a fluorescence marker for *E. coli* detection and quantification, and the incorporation of glutaraldehyde as a crosslinker between membrane amino groups and amino-functional quantum dots shortened the reaction time to 2 h at a bacterial concentration of up to 10^7^ CFU/mL [8]. In addition, Li* et al.* [10] have developed a matrix-assisted laser desorption/ionization mass spectrometry method for the identification of bacteria in water samples by loading filtration membranes with vancomycin-conjugated magnetite nanoparticles. Moreover, sugar molecules attached to magnetic iron oxide nanoparticles were used to isolate up to 88% of *E. coli* after incubation for 45 min and later detected by fluorescence staining [33]. Thus, the alteration of the original HAP-S structure and its functionalization with a nutritive mixture, which also includes sugar (HAP-S/CCL), leads to a substantial reduction in the detection time of *E. coli* (Figure 3) in comparison with HAP-S nanoparticles loaded only with the MUG substrate (from 3.30 to 1.30 h in the presence of *E. coli* at 10^8^ CFU/mL). At inoculum concentrations less than 10^8^ CFU/mL, HAP-S loaded with a high MUG content was not able to show fluorescence indicating substrate cleavage, even after 24 h. This result indicated that the MUG concentration in the ceramic matrix alone was not enough to visualize the glucuronidase activity. In general, it was demonstrated that the fluorescence occurs as expressed by Silbert* et al.* [34], in a shorter time than the visual detection of bacterial colonies in agar plates.

### 2.6. Activation (Hydration) of HAP-S/CCL before Detection of Bacteria

Bacterial adhesion to the nanoparticle surface is also related to the type of bacteria and matrix hydrophobicity [25]. Bacteria adhere differently depending on several factors reviewed previously. HA possesses both adsorption and absorption properties. On the other hand, taking into account the solubility of CCL and the HAP-S/CCL particle size dispersion in water, hydrating the composite before it comes into contact with bacterial cells might guarantee better availability of both nutrients and substrates.

**Figure 4:**
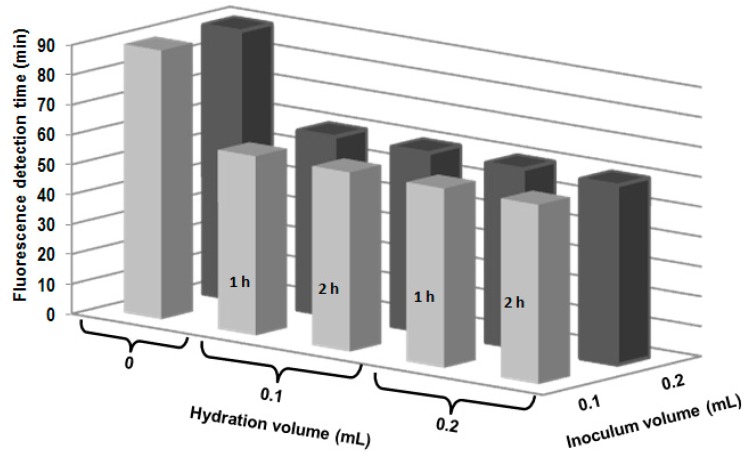
Hydration of HAP-S/CCL. Influence of the volume of water added (0.1 and 0.2 mL), hydration time (1 and 2 h) and inoculum volume (0.1 and 0.2 mL) on the fluorescence detection time.

The results of the influence of the pre-hydration with water added (0.1 and 0.2 mL) at hydration time (1 and 2 h) are shown in the Figure 4. Hydration of the HAP-S/CCL composite before inoculation with the bacterial suspensions showed that the hydration step reduced the fluorescence detection time from 90 to 60 min. However, no differences in detection times were detected when the HAP-S/CCL composite was hydrated with 0.1 or 0.2 mL of deionized water for 1 or 2 h with an inoculum volume of 0.1 or 0.2 mL. All the samples tested showed strong fluorescence.

The results of this test are consistent with those obtained in the previous experiment. The hydration procedure positively influenced the fluorescence reaction time, diminishing it to 60 min (Figure 4). The fluorescence reaction time did not depend on the hydration time or the water volume, possibly owing to the crystalline nature of the HAP-S nanoparticles and the water. Intense fluorescence was observed by the naked eye. Furthermore, hydration can influence the peptidoglycan membrane permeability of *E. coli* [10], thus allowing the faster exchange of enzyme and substrate through the membrane. In previous research with a chromogenic-fluorogenic liquid medium, *E. coli* was detected by fluorescence derived from MUG cleavage in water samples only after incubation at 35 ± 2 °C for 18–24 h [19]. Thus, our proof-of-concept procedure detected *E. coli* much faster (in only 1 h).

### 2.7. Fluorescence Detection with Different Inoculum Volumes and Concentrations

**Figure 5:**
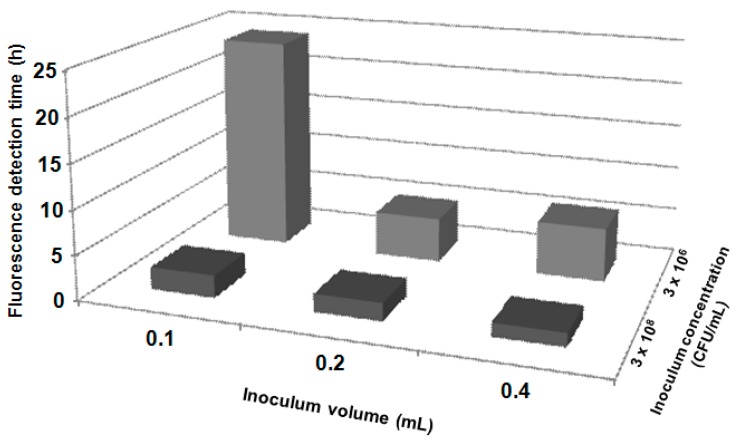
Influence of the *E. coli* inoculums volume (0.1; 0.2 and 0.3 mL) and concentration (3 × 10^6^ and 3 × 10^8^ UFC/mL) on fluorescence detection time.

Figure 5 describes the dependence of the inoculum volume and concentration on the visualization of the MUG cleavage reaction by β-glucuronidase of *E. coli.* With greater concentrations and volumes of the bacterial suspension, the visual fluorescence detection was faster and more intense. After the addition of 0.1 mL of *E. coli* suspension (3 × 10^6^ CFU/mL) to HAP-S/CCL composite, fluorescence was not detected by the naked eye after incubation at 35 ± 2 °C for 6 h. In this case, the reaction was visible only at 24 h. Increasing the amount of sample (suspension) up to 0.4 mL did not accelerate the detection to less than 3 h. Only HAP-S/CCL samples with an inoculum concentration of 3 × 10^8^ CFU/mL allowed a fast detection period of 2–2.5 h, with an inoculation volume of 0.1 or 0.2 mL; the detection period decreased to 90 min with an inoculation volume of 0.4 mL.

Silbert* et al.* [34] tested a procedure to detect *Salmonella enterica* serovar Typhimurium 1a bacteria with nanoparticles in 10 h at an inoculum concentration of 10^7^ CFU/mL. A much lower detection limit (from 10^2^ to 10^7^ CFU/mL) and faster fluorescence observation was described by Fu* et al.* [8], who used CdSe/ZnS/SiO_2_ composite fluorescent quantum dots and 80-min incubation. With a greater concentration of inoculated bacteria, our HAP-S/CCL composite produced a shorter response time at a given inoculum volume (Figure 5). The minimum observed detection time (60–120 min) for these proof-of-concept experiments did not exceed the expected limits described by Silbert* et al.* [34], who used a platform for visual detection of bacteria based on the interaction of membrane-active compounds secreted by bacteria with agar-submerged nanoparticles comprising phospholipids and the chromatic polymer polydiacetylene.

### 2.8. Detection of E. coli Fluorescence by the Spectroscopic Method

With the proof-of-concept experiments for the fluorescence detection of *E. coli*, we expected to reduce the time interval for detecting the fluorogenic reaction. Some spectroscopic methods [7,34] that have been developed for rapid bacterial detection (e.g., for *S. typhimurium* and other bacteria with immunomagnetic, immunofluorescent, or lipid-coated nanospheres) were able to detect target microorganisms in a range from 10^5^ to 10^7^ CFU/mL by fluorescence using a spectrometer. These results confirmed that the time required to detect the reaction could be significantly reduced.

**Figure 6:**
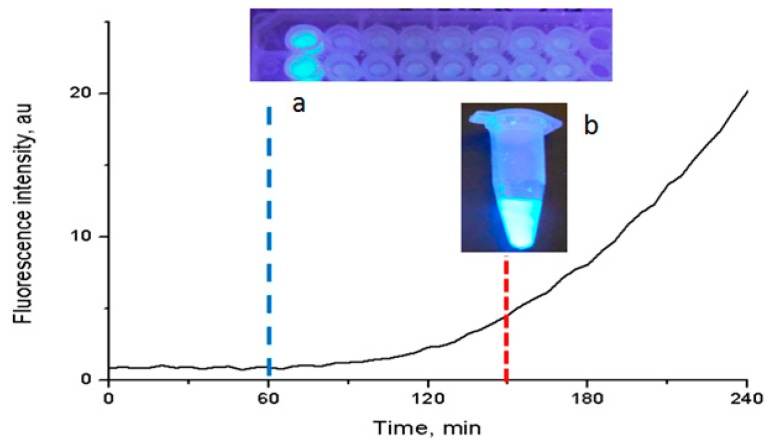
Spectroscopic and visual detection of the fluorogenic signal after 1 to 6 h of incubation time. (**a**) Image of the fluorescence spectrofluorometric detection in a microplate. (**b**) Image of the visual detection in an Eppendorf tube.

The proof-of-concept spectroscopic detection of the fluorogenic signal, derived by MUG substrate cleavage in the presence of the HAP-S/CCL composite, is illustrated in Figure 6a. The graph corroborates the results obtained when the reaction was detected by visual observation under UV light (Figure 6b): a well-defined release of the fluorogenic compound methylumbellipheryl started at 60 min. The rate of fluorescence increased after incubation at 35 ± 2 °C for 90 min. The fluorescence due to substrate cleavage was visible with high intensity after incubation for 150 min.

In our experiments, the reaction detection time was reduced from 150 min, when fluorescence was detected by the naked eye, to 60 min when the fluorescence intensity was measured using a fluorescence spectrophotometer. These results show a high sensitivity at a high bacterial concentration (10^6^–10^8^ CFU/mL). However, other studies are needed to establish the detection limit and to test other Gram-negative and Gram-positive bacteria suspensions, as well as artificially spiked or actual clinical samples. In the near future, the development of inexpensive, easy to handle nanodiagnostic devices could be used at the point-of-care to implement timely personalized disease detection and prevention methods.

HA is a widely available and not highly expensive natural starting material for structuring nanocomposites. The main advantages of using solid nanocomposite diagnostic devices instead of liquid devices for bacterial testing include not only prolonged shelf life and stability, but also easier handling, packaging and simplicity of testing procedures.

## 3. Experimental Section 

### 3.1. Materials

The substrate MUG was from Applichem, Darmstadt, Germany. Tryptone soy agar (TSA), chromogenic-fluorogenic medium for isolation and rapid identification of *E. coli* and coliforms (CCL), brain heart infusion broth (BHI), and Mueller-Hinton Agar (MHA) were obtained from BioCen, Bejucal, Cuba. Antibiotic disks (gentamicin, amikacin, sulfamethoxazol-trimethoprim, and chloramphenicol) were purchased from Oxoid Ltd. Inc. (Hampshire, UK). All calcium phosphate samples (HAP-S) were obtained by modification of the starting calcium carbonate source through an ionic exchange thermo chemical reaction and calcium carbonate was transformed to calcium phosphate. HAP-S samples were kindly donated by the Centro Nacional de Investigaciones Científicas(La Habana, Cuba). All HAP-S samples were sterilized in autoclave at 121 °C for 15 min before use.

Bacteria strains were obtained from the culture collection of the Centro Nacional de Biopreparados, Bejucal, Cuba (*E. coli* ATCC 25922, *Pseudomonas aeruginosa* ATCC 27853, *Staphylococcus aereus* ATCC 25923, *Enterococcus faecalis* ATCC 29212). Pure strains were activated in brain heart infusion broth (BHI) in an incubator at 35 ± 2 °C for 24 h. Required volumes of BHI broth were transferred into sterile tubes. Saline solution (0.85% w/v) was added and the suspension was homogenized in a vortex mixer. The bacterial cell concentration was adjusted to 75% transmittance at 580 nm wavelength (approximately to 3.0 × 10^8^ CFU/mL) in an spectrophotometer (T70 UV/VIS Spectrometer, PG Instruments Ltd., Lutterworth, UK).

### 3.2. Preparation of the HAP-S/CCL Composite

HAP-S and CCL were selected for the preparation of the nanocomposite (HAP-S/CCL). Briefly, CCL was suspended in deionized water (23.9 g/L) and heated to boil for 2–3 min. HAP-S nanoparticles were loaded into the nutritive mixture with the fluorogenic substrate MUG for up to 3 h at 25 °C, and the excess of water was evaporated in a constant velocity laminar air flow at 25 °C for 3 h (Faster BH-EN 2004, Bardissi Medical, Cairo, Egypt) or in a vacuum-drying oven at 60 °C for 3 h (Heraeus, Hanau, Germany). The CCL:HAP-S ratio was set at 2.5:1 (v/w).

### 3.3. Loss on Drying Test

The loss on drying of samples was calculated by weighing the samples from 0.2 to 0.4 g (*n* = 3), drying the samples at 105 °C for 4 h in a drying oven with air circulation, and calculating the values as described in the 35 US Pharmacopeia [35].

### 3.4. Loading Capacity Test

To determine the loading capacity (LC) of HAP-S nanoparticles, 0.2–0.3 g samples (*n* = 3) were weighed and then submerged in 3 mL of deionized water for 1–3 h. After this period, the excess water was eliminated by decantation and extraction of the supernatant. The loss on drying of samples was performed as described previously. The LC was calculated according to the following equation:

LC = LDAS − LDBS (%)
(1)

where LDAS is the loss on drying (%) of the HAP-S nanoparticles after submersion in deionized water, and LDBS is the loss on drying (%) of the HAP-S nanoparticles before submersion in deionized water.

### 3.5. pH Determination

The HAP-S nanoparticles were mixed with deionized water for 15 min in a blender, and the pH value was measured by a potentiometric method (Radiometer pH meter, Copenhagen, Denmark) at 25 °C. CCL was dissolved in deionized water (2.39 g/100 mL) and the pH was measured directly on the broth at 25 °C.

### 3.6. Characterization of the HAP-S and HAP-S/CCL

The surface characteristics of the HAP-S nanoparticles and the HAP-S/CCL composite (e.g., roughness, granular morphology, porosity, and aggregation) were studied by scanning electron microscopy (SEM) (Jeol JSM, Model 6360LV, Tokyo, Japan). Images were processed with Image J software (National Institutes of Health, Bethesda, MD, USA). These parameters were measured at the Microscopy Center of the Federal University of Minas Gerais (MC-UFMG).

The Zeta-potentials (ζ-potentials) of the elemental nanoparticles of HAP-S, CCL, and the HAP-S/CCL composite were measured by dynamic light scattering using a Zetasizer, Nano Series (Malvern Instruments Ltd., model Nano ZS, Worcestershire, UK). Aggregated HAP-S nanoparticles were vortexed in water at 25 °C for 30 min. to disaggregate them. The particle size (the average from quintuplicate measurements) and percentage distribution were also measured and calculated using the Zetasizer. A temperature of 37 °C was selected for the measurements as it corresponds to the incubation temperatures for most bacteria.

### 3.7. Determination of the Possible Antibacterial Activity of HAP-S

In order to evaluate the possible antibacterial activity of the HAP-S nanoparticles, different HAP-S suspensions (0.05%, 0.5%, 1%, 5%, 10%, 15%, and 20% (w/v)) were placed on 15-cm Petri dishes containing MHA. Agar layer was concentrically perforated (5 mm) to allocate HAP-S suspension samples. Plates were previously inoculated by the spread plate method with standardized suspensions (Tube No. 1 McFarland Scale, bioMérieux, Marcy l’Etoile, France) of *E. coli* ATCC 25922, *Staphylococcus aureus* ATCC 25923, *Pseudomonas aeruginosa* ATCC 27853 and *Enterococcus faecalis* ATCC 29212. Plates were incubated at 35 ± 2 °C for 24 h. The presence or absence of inhibition halos (and their diameters) were observed. As controls, disks containing gentamicin (for *E. coli*), amikacin (for *P. aeruginosa*), sulfamethoxazole-trimethoprim (for *E. faecalis*), and chloramphenicol (for *S. aureus*) were placed on the surface of the medium.

### 3.8. Role of the Nutritive Mixture on the Fluorescence Detection of Bacteria

In order to evaluate the role of the nutritive mixture on the fluorescence detection time, two different composites were formulated, one with the nutritive-fluorogenic substrate mixture (HAP-S/CCL) containing nutritive bases and other growth promotion ingredients as well as the fluorescent substrate MUG, and a second one (HAP-S/MUG) without the nutritive mixture but with the fluorescent substrate MUG. Three different bacterial concentrations were examined (3 × 10^8^, 3 × 10^6^, and 3 × 10^4^ CFU/mL), and each of them was added to the composites at two concentrations (0.25 and 0.5 g/mL) to 0.1 g samples.

The MUG solution was prepared in sterile deionized water (0.02 g/L) and sterilized by filtration (0.2 μm pore size). HAP-S (1 g) was heated in an oven at 180 °C for 1 h. The HAP-S/CCL composite was obtained by mixing 1 g of HAP-S and 2 mL of 1:1 solution composed by CCL (23.6 g/L) and MUG (0.02 g/L). The HAP-S/MUG composite (CCL was substituted by MUG) was formulated by mixing 1 g of HAP-S with 2 mL of MUG solution. The resulting HAP-S/CCL and HAP-S/MUG composites were dehydrated under a laminar airflow for 4 h. The *E. coli* suspension was standardized to 3 × 10^8^ CFU/mL in sterile saline solution. Next, the samples were incubated at 35 °C and fluorescence (under 366 nm light with a UV lamp) was detected by visual observation every 30 min up to 6 h and then again after a 24-h incubation period.

### 3.9. Activation (Hydration) of HAP-S/CCL before Detection of Bacteria

An standardized suspension of *E. coli* (3 × 10^6^ CFU/mL; 0.1 and 0.2 mL) was added to the HAP-S/CCL composite (0.25 or 0.4 mL). All samples were incubated at 35 ± 2 °C for 3 h, and fluorescence was visually observed at 1-h intervals under UV light (366 nm).

### 3.10. Fluorescence Detection with Different Volumes and Concentrations of Inoculums

An standardized suspensions of *E. coli* (3 × 10^8^ and 3 × 10^6^ CFU/mL; 0.1, 0.2, and 0.4 mL) was added to the HAP-S/CCL composite (0.1 g). Samples were incubated at 35 ± 2 °C for 3 h, and fluorescence was visually observed at 1-h intervals under UV light (366 nm).

### 3.11. Detection of E. coli Fluorescence by a Spectroscopic Method

Fluorescence detection was carried out using a spectrophotometer (Varian, Cary Eclipse, Victoria, Australia) at an excitation wavelength of 460 nm and an emission wavelength of 366 nm. A standardized suspension of *E. coli* (3 × 10^8^ CFU/mL; 0.2 mL) was added to the HAP-S/CCL composite (0.1 g), and the mixture was incubated at 35 ± 2 °C for up to 4 h. In parallel, other samples of the HAP-S/CCL composite were placed in the *E. coli* suspension and were incubated under the same conditions; then, the fluorescence was visually observed at 30-min intervals under UV light (366 nm).

### 3.12. Statistics

All statistical analyses such as analysis of variance, the Tukey post-hoc test, and descriptive statistics (median, standard deviation) were executed with Statistic 8 software (Statsoft Inc., Tulsa, OK, USA).

## 4. Conclusions

In summary, we characterized calcium phosphate nanoparticles and aggregates, and demonstrated their suitability for obtaining a device that detects *E. coli* by combining the HAP-S matrix with a highly nutritive mixture and a specific fluorogenic substrate. The presence of bacteria could be identified by either visual or spectrofluorometric detection methods. The method described herein shows several advantages over current bacterial identification procedures: (a) rapid detection time; (b) simplified workflow; and (c) inexpensive, commercially available reagents and components. Thus, the developed calcium phosphate nanocomposite may be useful as a model for the development of other nanoparticle composites for bacterial detection.
